# Youth perceptions of Brand variant names on standardised cigarette packs, and responses to replacing these with numbers: a focus group study in Britain

**DOI:** 10.1080/09687637.2021.1902479

**Published:** 2021-05-26

**Authors:** Danielle Mitchell, Crawford Moodie, Allison Ford, Anne Marie MacKintosh, Nathan Critchlow, Linda Bauld

**Affiliations:** a Institute for Social Marketing and Health, Faculty of Health Sciences and Sport, University of Stirling, Stirling, UK; b Usher Institute, College of Medicine and Veterinary Medicine, University of Edinburgh, Edinburgh, UK; c SPECTRUM Consortium, University of Edinburgh, Edinburgh, UK

**Keywords:** Adolescent smoking, Tobacco control, Tobacco packaging

## Abstract

Tobacco companies use brand variant name on cigarette packaging to differentiate, and create interest in, their products. We explored young peoples’ reactions to brand variant names on cigarette packs and perceptions of replacing these with numbers, a proposed policy in Turkey. Twelve focus groups, segmented by gender, age (11-12, 13-14, 15-16) and social grade (ABC1, C2DE), were conducted across Britain from May–July 2018 (*n* = 89). Participants were asked what they thought about brand names in general, and on cigarette packs, and perceptions of replacing the brand variant name on cigarette packs with a number. Brand (variant) name was considered important for products, including cigarettes, and thought to communicate information about the product, image, price, and taste, and encourage purchase. Although replacing brand variant names on cigarette packs with numbers caused confusion, several participants mentioned that it would eliminate any remaining marketing power that the pack may have. They thought that numbered cigarette packs could be off-putting due to the absence of a familiar brand name, although the impact on smokers was considered negligible. Although adolescents were not clear on the rationale for numbered cigarette packs, some suggested that this would reduce one of the few remaining promotional features on standardised packs.

## Introduction

Tobacco marketing directly influences smoking uptake, use, and addiction, and therefore contributes to smoking-related morbidity and mortality (Henriksen, 2012). In response, many countries have introduced comprehensive bans on tobacco advertising, promotion, and sponsorship, including the open display of tobacco products in retailers (Drope et al., 2018; He et al., 2018). In addition, more than 100 countries require large pictorial warnings (covering at least 50% of the main display areas) on packs (Canadian Cancer Society, 2016) and at least 14 countries now require cigarettes to be sold in standardised (or plain) packs (Tobacco free kids, 2020). Consequently, in many countries, one of the remaining ways that tobacco companies can still promote their products is through brand and variant name.

Brand variant names are important to tobacco companies as they help to sustain or increase brand awareness, enable differentiation within a brand portfolio, signal product quality, appeal to different consumer segments, and indicate taste and flavour (Doxey & Hammond, 2011; Friedman & Dipple, 1978; Hammond et al., 2013; Skaczkowski et al., 2017). These are all factors that contribute to constructing and maintaining positive brand equity (Aaker, 2009; Aktaş Arnas et al., 2016; Hafez & Ling, 2005; Yoo et al., 2000). Research with female youth and adolescents, for instance, found that brand names such as Vogue, Silk Cut and JPS Legendary Black were appealing, even on standardised packs, suggesting that name alone can create a positive brand image, e.g. fashionable, glamorous or coolness (Hammond et al., 2013; Mitchell et al., 2019).

In recognition of the promotional role of brand variant name, and to limit it being used as an incentive to smoke, the Turkish Government proposed that it be removed from cigarette packs and replaced by a number (Sigara Yasağında Yeni Dönem & Hurriyet, 2014). The numbers would be assigned alphabetically to all brand variants on the market at the time of the legislation; for example, in the UK American Spirit Blue would be ‘1’, American Spirit Orange ‘2’, Benson & Hedges Blue ‘3’, and so forth, with brand variants launched thereafter assigned the next highest available number (Moodie, 2016; Mucan & Moodie, 2018). The proposed change would permit tobacco-selling retailers to carry a list showing the number and corresponding brand variant to enable consumers to identify the product they wished to purchase, similar to the price lists currently allowed in countries that have banned the open display of tobacco products in retailers (Eadie et al., 2016; Moodie, 2016; Mucan & Moodie, 2018). The brand variant name would not, therefore, be banned by government, it would just not be permitted for tobacco companies to display it on packs.

Only one study, qualitative research with young adult (18–24 years) smokers in Turkey, has explored reactions to ‘numbered’ standardised cigarette packs (Mucan & Moodie, 2018), with participants viewing the brand variant name to be important and numbered packs as having the potential to reduce appeal. By removing the brand variant name from the pack, they thought that non-smokers or newer smokers could be deterred as it may be confusing, and they be unable to distinguish different brands or form positive brand images (Mucan & Moodie, 2018); brand image is important to both tobacco companies and consumers (Hastings & MacFadyen, 2000).

No research has explored the potential impact of ‘numbered’ packs in a country with standardised packaging, which has been required for cigarettes sold in the UK since May 2017 (UK Government, 2015), or among adolescents. As brand variant name is arguably the last remaining promotional feature on standardised packs, and past research shows that cigarette brand variant names can appeal to adolescents, a key target population for preventing uptake, we extend past research by exploring adolescents’ perceptions of cigarette brand variant name and response to numbered packs.

## Methods

### Design and sample

Twelve focus groups were conducted with 11-16 year olds (*n* = 89) in Scotland (Glasgow), England (Newcastle) and Wales (Cardiff) between May and July 2018, one-year post-standardised packaging (UK Government, 2015). These locations were selected to ensure participants were drawn from each of the three countries in urban, easily accessible locations. Groups were segmented by age (11-12, 13-14, 15-16) due to the extent to which young people develop between the ages of 11 and 16, gender, and social grade (ABC1, C2DE), with up to eight participants in each group (Table 1). Social grade was determined by the occupation of the primary income earner of the household, with grades A, B and C1 signifying those in a higher social grade and grades C2, D and E those in a lower social grade (National Readership Survey, undated). We did not attempt to segment the sample by smoking status given very low rates of smoking among younger people and particularly the youngest groups. Participants were recruited in friendship pairs by professional market recruiters in each location. Participants were reimbursed to the value of £15 as a thank you for their time and contribution.

**Table 1.** Sample demographics.

Group	Gender	Age	Social Grade	Country
1	Male	11-12	ABC1	Scotland
2	Female	11-12	C2DE	England
3	Female	11-12	ABC1	Wales
4	Male	11-12	C2DE	Wales
5	Female	13-14	ABC1	England
6	Male	13-14	C2DE	Wales
7	Male	13-14	ABC1	England
8	Female	13-14	C2DE	Scotland
9	Male	15-16	ABC1	Scotland
10	Female	15-16	C2DE	Scotland
11	Female	15-16	ABC1	Wales
12	Male	15-16	C2DE	England

### Materials

**Figure 1.**
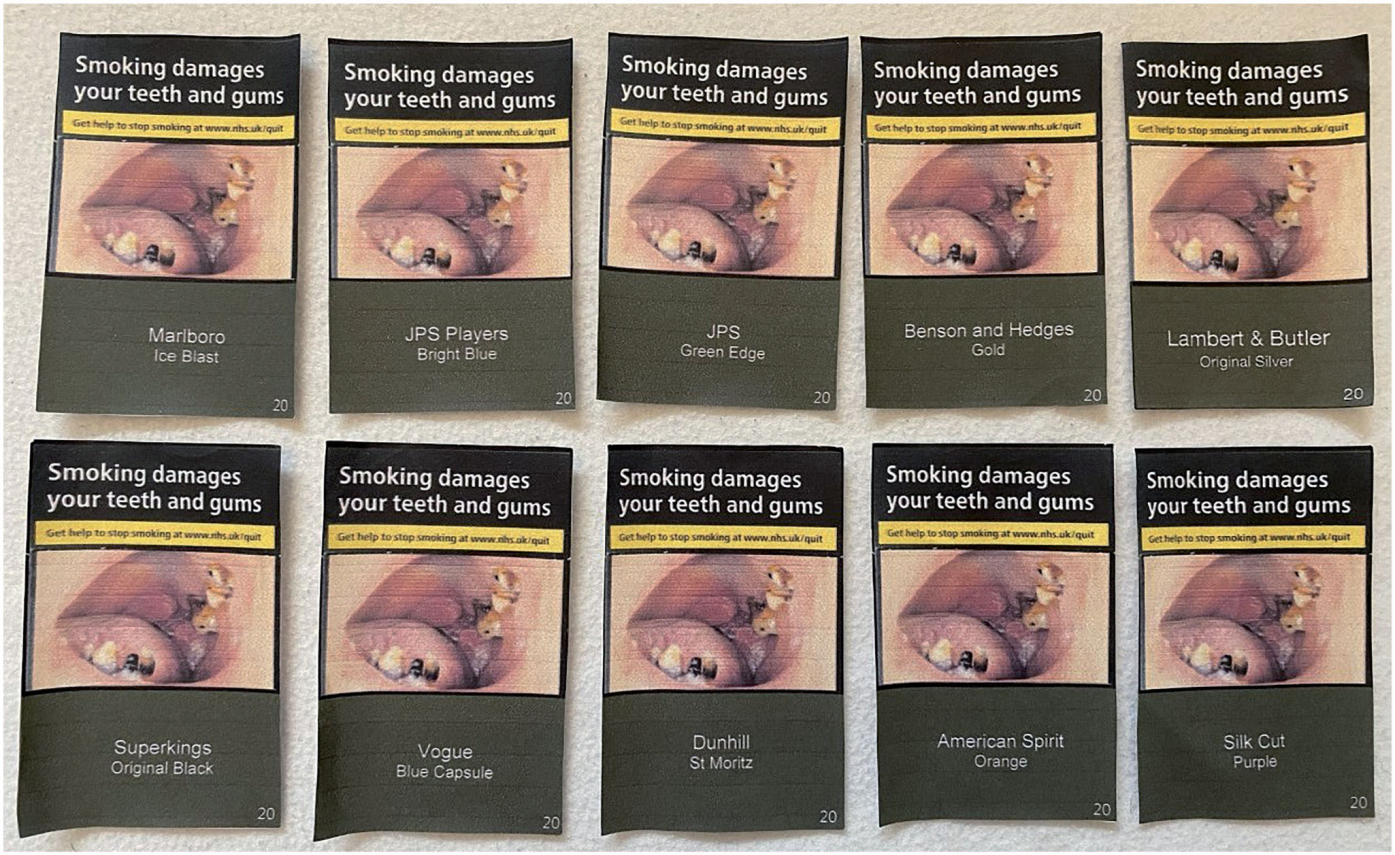
Images of cigarette packs with brand variant name.

**Figure 2.**
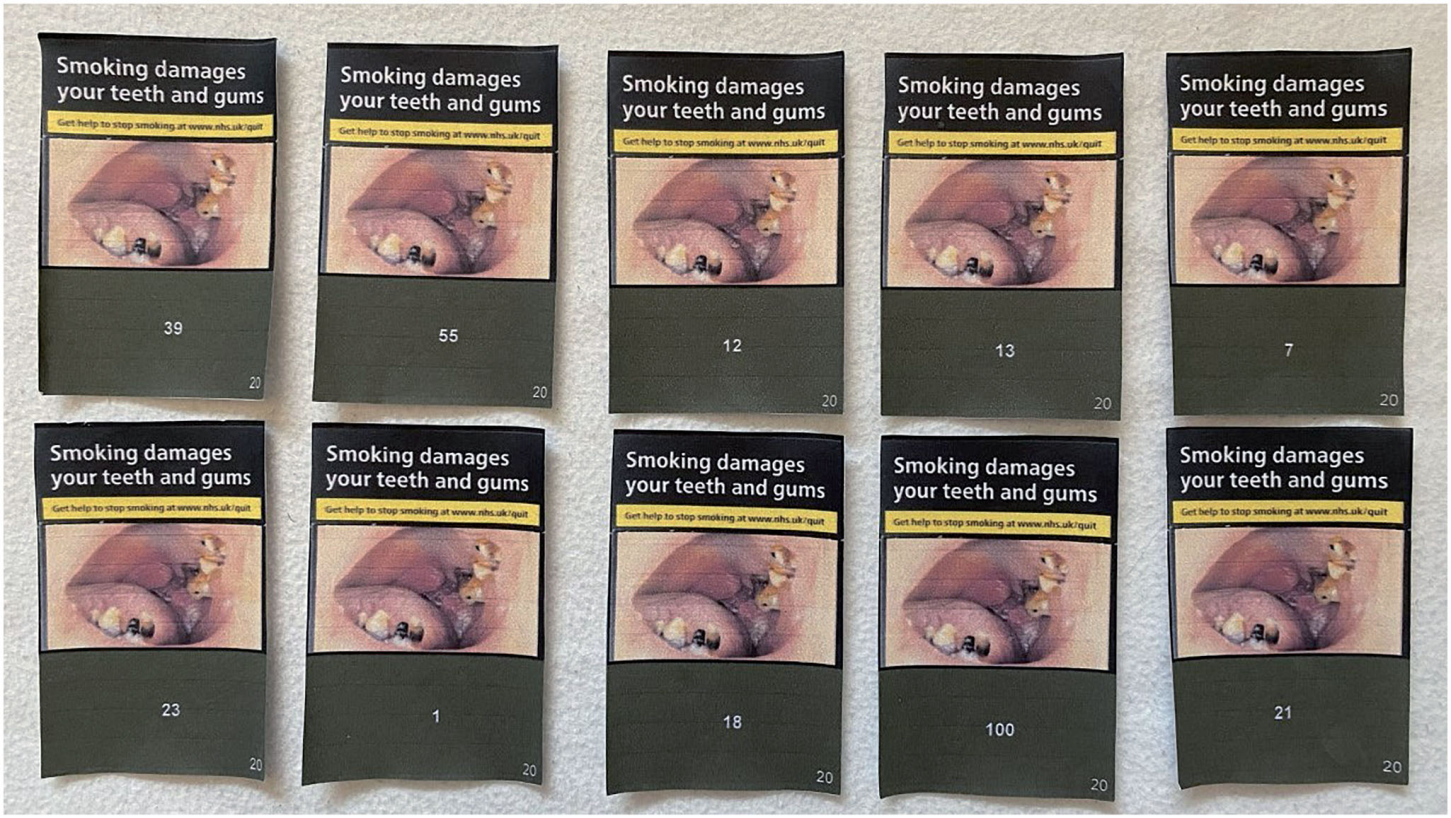
Images of cigarette packs with numbers.

Participants were shown 20 images of standardised cigarette packs, with 10 showing packs with different brand variant names and 10 showing packs with different numbers. The images were designed to be a similar size to cigarette packs (Figure 1). An image of a front facing UK standardised cigarette pack as displayed on supermarket websites (e.g. Tesco) was used as a template for the images, with this providing a simple design that was easily replicated to show a range of brand variant names and numbers. The brand variants used were chosen to show that there are different brands available in the UK and a range of variant names, many of which include colour and/or product descriptors (e.g. American Spirit Orange, Vogue Blue Capsule) (Figure 1). The numbers were chosen, by DM and AMM, to explore how adolescents reacted to them and what connotations were drawn, if any. This included potentially lucky or superstitious numbers (e.g. 7 and 13), those that may deemed appealing or memorable (e.g. 1 and 100), and numbers selected at random (e.g. 12 or 39) (Figure 2).

**Figure 3.**
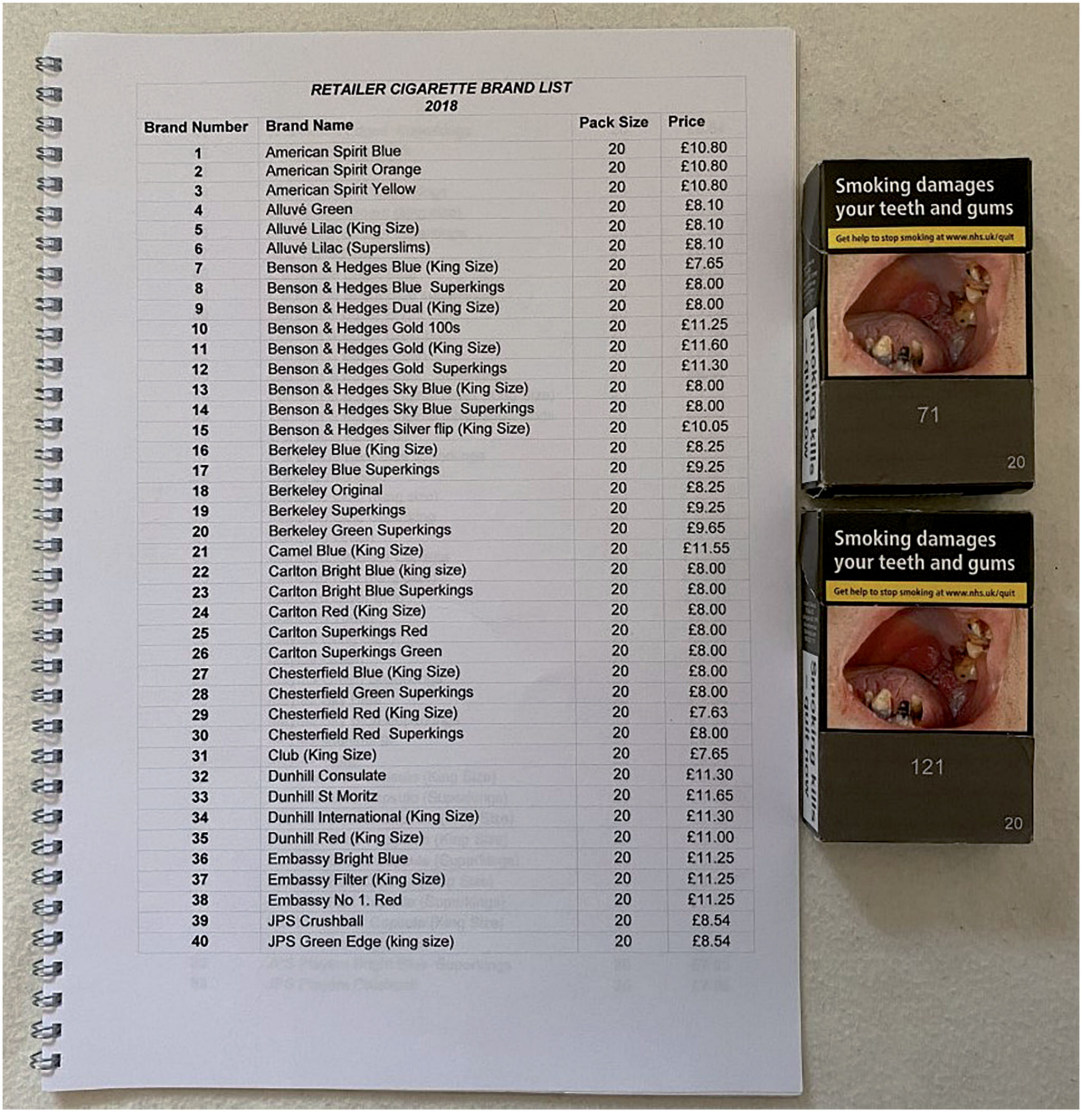
Example price list and mock packs.

We also modified a ‘regular’ standardised pack to show a randomly selected number rather than brand variant name, and a mock brand list (which included all cigarette brand variants on the UK market at the time of the study) showing which number corresponded with which brand variant, in alphabetical order, as a prompt to help participants understand the concept. The mock pack and list were used to show participants how the measure may work in shops where tobacco is sold (Figure 3).

### Procedure

All groups were moderated by two researches (AF/DM or AMM/DM). The section reported in this paper was led by DM. The discussion followed a semi-structured topic guide. At the start of each group, we explained to participants that we were interested in understanding how they view tobacco marketing. They were informed of the format of the discussion, and reminded that the group was voluntary, confidential and would be audio recorded. As a warm up activity, participants were asked about their general shopping behaviour. They were then asked about their knowledge of, and reactions to, standardised cigarette packaging (findings not presented here), before being asked about brand variant names and their reactions to numbered cigarette packs, with this section lasting, on average, 10 minutes.

Throughout the focus group, participants were encouraged to engage with a range of stimuli laid out in front of them. This included participants handling, sorting and grouping tobacco packs within the first component of the discussion and for the section on brand variant name and numbered packs, reported here, participants were asked to organise, if possible, 20 images of packs. This approach was used as a means of encouraging discussion and understanding participants’ unprompted response to the named and numbered packs. Once participants had grouped the images, they were asked to explain why they had done so and whether there was anything they noticed about the images. Participants were asked about the different brand variant names and numbers, if they knew of any commercial brands other than cigarette companies (e.g. general products that they purchase) that used numbers in their brand (or brand variant name) and whether, in general, they thought that the brand name is important, and if so why. The concept of numbered packs was then explained to the participants, with the price list and mock packs shown to illustrate how the measure would work in practice. In six groups, mostly younger groups (11-12 or 13-14 years) who struggled to understand the concept when it was explained to them, the example (price list and mock packs) was not shown as we felt this would cause further confusion. Participants were asked why they thought this idea may have been suggested and why tobacco companies use different variant names (if this had not come up previously). Finally, they were asked for their thoughts on how non-smokers and smokers would respond and we asked for their views on whether this was a good or bad idea. The second moderator took notes during this section and observed participants’ verbal and also non-verbal responses to the images to help understand their reactions.

### Ethics

Ethical approval was obtained from the General University Ethics Panel at the University of Stirling (GUEP420). Participant and parent information sheets and GDPR forms were provided prior to the groups. Written informed consent was obtained from participants and a parent or guardian. At the start of each group, participants were reminded that the study was confidential, their responses would be anonymous, and that they were not obliged to answer any questions. At the end of each group, participants were provided with a leaflet about the harms of smoking and sources of further information.

### Analysis

The audio recordings were transcribed by external transcribers contracted by the University of Stirling. All transcript sections pertaining to brand variant name and numbered packs were checked against the audio recordings to ensure accuracy by DM, who conducted the analysis. Data were analysed using thematic analysis using both a deductive and inductive approach (Braun & Clarke, 2006). DM read the transcripts multiple times to familiarise herself with the content and identify possible themes. The analysis was facilitated by NVivo 11. Initial themes were developed into a thematic coding framework by DM, and then developed, labelled and interpreted until a consensus was reached within the research team (AMM, AF, CM and NC).

## Results

### The meaning, and communicative power, of Brand names

The consensus was that brand name is important for people in general, and for them personally. One reason was the belief that others may form an image of them based on, for instance, the brands they wear, given that brand name can communicate the price of a product. The youngest (11-12) male groups commented that brand name enables differentiation, e.g. ‘*It* [brand name] *separates that brand from other brands*’ (Males 11-12 ABC1, Scotland). Some participants agreed that brand name mattered but were unable to offer a rationale as to why, with this most common among females.

*Sometimes like a brand name would be like… for instance like North Face* [outdoor wear brand], *Armani* [designer fashion brand] *… quite expensive stuff* (Males 11-12 ABC1, Scotland)

*It makes people have an opinion on you, if you wear cheap clothes… if you wear expensive clothes* (Males 16-17 ABC1, Scotland)

Perceptions of the cigarette brand variant names usually occurred through facilitator prompts to look at, or think about, specific names, rather than organically. This may have been, at least in part, because participants were typically drawn to the pictorial warnings on packs.

***What about Silk Cut Purple? (moderator)***

*I like purple.*

*Does that communicate anything to you? No?*

*I don’t pay attention to the names, just the pictures.* (Males 13-14 C2DE, Wales)

***So what’s the first thing you notice about these images? (moderator)***

*The picture* (Female 13-14 ABC1, England)

When discussing brand variant names, Marlboro Ice Blast reminded the majority of participants of iced slushy drinks, including specific mention of Tango Ice Blast (a sugary slushy drink), with it suggested that the cigarettes would be menthol or fruity in flavour, e.g. ‘*It’ll be menthol*’ (Females ABC1 13-14, England). For American Spirit Orange, some of the youngest participants (11-12) suggested that it sounded like an alcoholic drink, soft drink, or fragrance. Vogue was consistently compared to fashion and the luxury fashion and lifestyle magazine, and considered popular, particularly among ABC1 females.

***What about American Spirit Orange? Have you ever heard of that one before, American Spirit?* (Moderator)**

*Sounds like an alcohol*

*It sounds like a spray*

*A fizzy drink* (Females 11-12 ABC1, Wales)

*If you saw that* [American Spirit Orange] *on drink, you’d know it was a cool drink* (Males 13-14 ABC1, England)

***Vogue for example, does that name, that brand name tell you anything about those cigarettes? (Moderator)***

*Popular*

*Isn’t it a fashion company?* (Females 13-14 ABC1, England)

Several older (15-16) adolescents, mostly ABC1 males, discussed how cigarette brand and variant names could invoke perceptions of product price, quality, and taste. For example, the view that Marlboro, Dunhill and JPS were expensive brands was driven by seeing prices in shops, but also by the name. It was also felt that impressions of quality could be achieved through colour descriptors, such as ‘Gold’, and impressions of taste through descriptors such as ‘Green’, which was thought to indicate that the cigarettes were menthol.

***Can I just ask something; how do you know if something is expensive or not because they’re covered up now in the shops so how do you know? (Moderator)***

*Just if you buy them*

*The wee leaflets that tell you* (Males 15-16 ABC1, Scotland)

*You can tell with the name, like Gold that sounds quite expensive, the same with Dunhill* (Male 15-16 ABC1, Scotland)

*I would say a lassie, because its menthol, because girls like menthol and all that.*

***How do you know that one is menthol? (Moderator)***

*Because it’s Green* (Males 15-16 ABC1, Scotland)

### Attachment to cigarette Brand variant name

There were mixed views, particularly among males, regarding the importance of cigarette brand variant name to smokers. Some females mentioned that smokers may initially be confused, but once they knew what number their brand was they would get used to the change. Among ABC1 males aged 13-14 and C2DE males aged 15-16 brand name, for smokers, was thought to carry meaning and something that they resonate with, and form an attachment to.

*Is important* [Brand name]? *I’d say yes, because you kind of resonate that with your favourite cigarettes* (Males 15-16 C2DE, England)

*Some people might have an attachment to their cigarette brand, or it might signify something* (Males 13-14 ABC1, England)

However, this view was not unanimous, with C2DE males aged 13-14 year and ABC1 males aged 15-16 years, viewing brand variant name as an insignificant element of the pack.

*I don’t think it’s that important because all it is is a name, and it takes up a bit of space on the packet* (Male 13-14 C2DE, Wales)

*Before when the packets were like that, the brand names were important to you, so they could stand out and more people would buy their cigarettes but now it’s just a name* (Male 15-16 ABC1, Scotland)

### Perceptions of the concept of, and rationale for, numbered packs

Participants often appeared bemused by the numbered packs, finding it odd, silly, confusing or pointless, particularly C2DE females, younger (11-12 years) participants and ABC1 males, e.g. ‘*It’s weird. It doesn’t look right*’ (Females 11-12 ABC1, Wales).

***Do you think it’s a good or a bad idea for…?* (Moderator)**

*Bad.*

***Bad, why do you think bad?***

*People will get confused.*

*Wasting time* (Females 13-14 ABC1, England)

It was suggested that consumers would have to remember the brand variant numbers, as would retail staff who may accidentally give customers the wrong product. While younger ABC1 males and females suggested the availability of the product list in shops would make the transition from brand variant names to numbers easier, by doing so it would mean that customers would still be exposed to brand variant names, thus questioning the value of replacing brand variant name with numbers in the first place.

*But you can easily just read it* [the product list], *it’s much easier* (Females 13-14 ABC1, England)

Regarding why a government would propose such a measure, several males alluded to it reducing the promotional power of the packaging, e.g. ‘*So it’s not advertising them*’ (Males 13-14 ABC1, England), by making all packs equal. While it was commented that people would become accustomed to the change, the removal of brand variant name was thought to help direct further attention to the already prominent pictorial warning.

*Yes, it’s a good idea. It makes all the packets the same* (Males 13-14 C2DE, Wales)

*It gets rid of the final thing on the packet, the actual eye catching… at the same time it’s not going to make much of a difference once they find out what number it is* (Males 15-16 ABC1, Scotland)

(You would). *literally just see the picture and the message and that is it* (Males 13-14 C2DE, Wales)

### How would people respond to numbered packs?

Several participants, in particular females, felt that smokers would be annoyed or confused if the brand variant name was removed as they would not necessarily know what cigarettes they had. In addition, males and older (15-16) ABC1 females tended to think that numbered packs would not have any impact on smokers, as the product is unchanged and, as a consequence of addiction, they would only be interested in the cigarette itself, e.g. ‘*The product is exactly the same*’ (Females 15-16 ABC1, Wales). In contrast, within several older (15-16) C2DE groups it was suggested that both smokers and non-smokers might be put-off as it is not what they are familiar with and it may be less appealing as the brand variant name provides smokers some reassurance.

*If they’re used to a specific brand they might not like any other ones, and they might not know which one they could be picking up* (Females 11-12 C2DE, England)

*Less people would buy it*

***So you think it might put them off?* (Moderator)**

*Yeah*

*It makes them feel like less powerful* (Females 15-16 C2DE, Scotland)

*I think you feel a lot more, like, confident in your cigarettes if they have a brand on, rather than just a number* (Males 15-16 C2DE, England)

*I think if you already smoke, and obviously they changed the packaging and that, I think you would still smoke, but if you don’t smoke I think now that they’ve done that, it will persuade you not to or it might become less appealing* (Males 15-16 C2DE, England)

## Discussion

Among our sample of 11-16 year olds from across Britain, we found that brand and variant name was generally considered a mechanism to communicate information about the product, image, price, and taste, and was capable of encouraging purchase. With respect to replacing cigarette brand variant names with a number, participants did not entirely understand the concept of, or need for, such an approach, but some felt that it could reduce appeal and weaken the power of the brand.

The findings suggest that cigarette brand variant names can appeal to young people, consistent with previous research (Hammond et al., 2013; White et al., 2012). Specific brand variants conveyed information about the product, such as cost, and associations with other products, such as drinks or fashion, echoing past research with adolescents (Mitchell et al., 2019; Scheffels & Saebø, 2013). For example, Marlboro Ice Blast was frequently compared to iced drinks and fruity flavours, and colours such as ‘Gold’ were thought to signal expensive brands. However, responses to brand variant name occurred only when participants were asked directly about this, and not organically. Instead, they were drawn to the pictorial warnings, consistent with eye-tracking research which found that adolescent never-smokers attended to pictorial warnings on cigarette packs more than the branding (including brand variant name), whereas daily smokers attended to the branding more than the pictorial warnings (Maynard et al., 2014).

There was uncertainty among participants about how people would respond to numbered packs, with some considering it pointless or confusing for smokers and retail staff. As the product list would be available in retailers and, as a result, brand variant names would still be visible to a certain extent, this was thought to make it easier for consumers and retailers. Nevertheless, the question was whether this continued, albeit reduced, visibility would decrease the appeal of cigarettes to established smokers, particularly once consumers knew the number of their usual brand variant. Some participants mentioned that it could limit the ability of tobacco companies to promote their products, making all packs look even more uniform, and that should brand variant name be replaced by numbers it may help further increase warning salience. In addition, some felt that people would be less able to establish any thoughts or feelings towards numbered cigarette packs, suggesting that this may help weaken the power of cigarette branding. Research in Turkey with young adult smokers found, similar to this study, that some participants thought that numbered packs would look more simplistic and may be off-putting for newer smokers (Mucan & Moodie, 2018).

In terms of limitations, while participants were shown images of 20 packs to ensure that they were exposed to different brand variant names and numbers but the same warning, this method lacks realism, with the exception of the mock packs used as a prompt to explain the concept, although these were not shown in all groups, specifically in the younger groups where there was confusion when the concept was explained. We only showed a limited sample of numbers and brand variant names. As the groups were recruited using friendship pairs this may have resulted in groupthink, with participants potentially shaping their views and answers based on those of their friends. While the sample comprised adolescents from three major cities in Britain, and accounted for age, gender and social grade, the findings cannot be generalised beyond this study. As this was only the second study to explore numbered cigarette packs, there is value in further exploring the concept with adult smokers in countries where standardised packaging is fully-implemented and where the policy is being considered, as the removal of brand variant name could be considered as part of future standardised packaging regulations. Further research exploring the role that brand variant name plays in markets where standardised packaging has been implemented would be of significant value (Mutti et al., 2011; Skaczkowski et al., 2017, 2018), e.g. on perceptions of harm, taste, quality and brand attachment. Future research would also benefit from using experimental methods, where participants are exposed to packs with different brand variant names or numbers.

In conclusion, adolescents generally considered brand name an important element of a product. While the most prominent feature of the standardised pack images was the pictorial warnings, with brand variant name failing to capture attention unless prompted, the name was nevertheless capable of communicating a lot of information about the product. While young people were uncertain about the value of numbered packs, replacing brand variant names on cigarette packs with numbers may have a role to play in further weakening the power of branding.
